# IFN-γ enhances the antitumor activity of attenuated *salmonella*-mediated cancer immunotherapy by increasing M1 macrophage and CD4 and CD8 T cell counts and decreasing neutrophil counts

**DOI:** 10.3389/fbioe.2022.996055

**Published:** 2022-09-29

**Authors:** Huimin Xu, Linghua Piao, Yundi Wu, Xiande Liu

**Affiliations:** ^1^ School of Life Sciences, Hainan University, Haikou, China; ^2^ Department of Physiology, Hainan Medical University, Haikou, China; ^3^ State Key Laboratory of Marine Resource Utilization in South China Sea, Hainan University, Haikou, China

**Keywords:** BCI, S.tΔppGpp, IFN-γ, neutrophil, macrophage, T cell

## Abstract

Bacteria-mediated cancer immunotherapy (BCI) inhibits tumor progression and has a synergistic antitumor effect when combined with chemotherapy. The anti- or pro-tumorigenic effects of interferon-γ (IFN-γ) are controversial; hence, we were interested in the antitumor effects of IFN-γ/BCI combination therapy. Here, we demonstrated that IFN-γ increased the tumor cell killing efficacy of attenuated *Salmonella* by prolonging the survival of tumor-colonizing bacteria *via* blockade of tumor-infiltrating neutrophil recruitment. In addition, IFN-γ attenuated *Salmonella*-stimulated immune responses by stimulating tumor infiltration by M1-like macrophages and CD4^+^ and CD8^+^ T cells, thereby facilitating tumor eradication. Taken together, these findings suggest that combination treatment with IFN-γ boosts the therapeutic response of BCI with S. tΔppGpp, suggesting that IFN-γ/BCI is a promising approach to immunotherapy.

## Introduction

Immunotherapy is a novel approach that exerts anticancer effects by reprogramming and increasing immunosurveillance and reducing cancer-mediated immunosuppression (Banerjee et al., 2018; Chuntova et al., 2018; Knochelmann et al., 2018; Ciardiello et al., 2019). Immunological checkpoint blockers (ICB) targeting CTLA-4, PD-1, and PD-L1 have been approved by the FDA for use in clinical trials for melanoma and non-small-cell lung cancer therapy. These drugs block cancer-mediated immunosuppression and activate cytotoxic T lymphocytes, which then kill tumor cells (Tumeh et al., 2014; Auslander et al., 2018; Binnewies et al., 2018; Cristescu et al., 2018). However, some cancers, such as pancreatic and ovarian cancers, are characterized by poor T cell infiltration, making them less sensitive to ICBs; also, low levels of tumor antigen mutations, possible triggering autoimmune diseases, are limitations of these drugs (Galon and Bruni, 2019).

Compared with ICBs, bacteria-mediated cancer immunotherapy (BCI) is specific to tumors, is less toxic to normal tissues, and has a broad spectrum of anticancer activity. BCI-induced tumor regression is not only mediated by tumor cell killing bacteria themselves, but also by host immune cells (neutrophils, macrophages, and CD4^+^ and CD8^+^ T cells) (Coley, 1891; Jacob, 2016; Guo et al., 2020). However, BCI alone cannot suppress tumor growth or metastasis completely; therefore, regulating the immune response to BCI should be combined with other therapies such as chemotherapy or radiotherapy to develop an integrated approach (Jia et al., 2007; Chen et al., 2017; Igarashi et al., 2018; Liang et al., 2019).

Interferon-γ (IFN-γ) plays a key role in cell-mediated adaptive immune response (Schroder et al., 2004). IFN-γ is used for the clinical management of diverse malignancies, including bladder carcinoma, colorectal cancer, ovarian cancer, and adult T cell leukemia due to its antiproliferative, antiangiogenic, and proapoptotic functions (Zaidi and Merlino, 2011). However, other reports suggest that IFN-γ has tumor-promoting activities, particularly in the context of failed attempts to treat melanoma (Schiller et al., 1996). Thus, IFN-γ seems to have two faces: anti- and pro-tumorigenic. BCI-induced enhancement of IFN-γ expression in response to bacteria accumulating in a tumor is significant and is associated with antitumor activity (Kim et al., 2015). However, relatively little is known about the antitumor effects of BCI/IFN-γ combination therapy. Therefore, the present study aimed to determine the effect of BCI/IFN-γ on antitumor and immune responses in a mouse model of colon cancer.

## Materials and methods

### Mouse colon cancer model and bacterial injection

SPF BALB/c male mice (five to six weeks old; 18–25 g) were purchased from Guangdong Medical Laboratory Animal Center (Foshan, China). All mouse experiments were supervised by the Animal Science and Technology Ethics Committee of Hainan University. Mice were anesthetized with either 2% isoflurane or ketamine (200 mg/kg). To generate colon cancer xenografts, CT26 cells (1 × 10^6^; ATCC) cultured in DMEM plus 10% FBS (Gibco, United States) were implanted into the right flank by subcutaneous injection. Tumors were measured every day using a caliper, and volume was calculated using the following formula (length × height × width)/2. Tumor-bearing mice were divided randomly into the following four groups (n = 9/group): PBS, IFN-γ (Z02916-100, Genscript, Nanjing, China), S. tΔppGpp, or S. tΔppGpp + IFN-γ. The IFN-γ groups received 10 mg/kg IFN-γ daily *via* intravenous injection. When the tumor volume reached 120–160 mm^3^, mice received an injection of 3 × 10^7^ colony-forming units (CFU) of S. tΔppGpp bacteria into the tail vein. When the tumor volume reached ≥1,500 mm^3^, mice were euthanized. Attenuated *Salmonella typhimurium*, St.△ppGpp (defective in the synthesis of ppGpp (*RelA::cat*, *Spot::kan*)) carrying the luciferase gene *Lux* (S.t△ppGpp-lux; SHJ2168) was obtained from J. J. Min (Institute for Molecular Imaging and Theranostics, Chonnam National University Hwasun Hospital, Jeonnam, Republic of Korea). Bacteria were cultured in Luria Bertani medium containing kanamycin (Sigma-Aldrich) and stored as 25% glycerol stocks at -80 °C.

### Optical bioluminescence imaging

Tumor-bearing mice were injected intravenously with 3 × 10^7^ CFU S. tΔppGpp prior bacterial bioluminescence imaging when tumor volume reached 120–180 mm^3^. Mice were divided into two groups (n = 6/group): S.t△ppGpp Lux and S.t△ppGpp Lux + IFN-γ (intravenous administration 10 mg/kg daily). Bacterial bioluminescence imaging was performed for 9 days using an IVIS 100 (Caliper).

### Bacterial counting

Tissues including tumor, liver, lung, and spleen were collected from tumor-bearing mice. Ground tissue was transferred to agar Petri dishes and incubated overnight at 37°C. The number of bacteria per Gram of tissue was calculated using the formula: Y × 10^Z^ × (1 + X) × 10/X (CFU/g; where X: tissue weight; Y: bacterial number on the Petri dish; Z: dilution factor).

### H&E staining

Heart, liver, spleen, kidney, and lung were removed from euthanized tumor-bearing mice and kept in 4% PFA solution for toxicity evaluation of S.t△ppGpp/IFN-γ combination therapy. Paraffin sections (3 μM) were stained with a Hematoxylin and Eosin Staining Kit (C0105, Beyotime) in accordance with the manufacturer’s protocol.

### Immunohistochemistry (IHC)

Tumors were removed from euthanized mice and kept in 4% PFA solution. Paraffin sections (3 μM) were incubated in QuikBlock™ Blocking Buffer (P0260, Beyotime), followed by exposure to Ly-6G/Ly-6c (Santa Cruz Biotechnology, Inc.), CD86, and CD206 antibodies (Boster Biological Technology Co., Ltd.) at 4°C. The sections were visualized with 3, 3′-diaminobenzidine tetrahydrochloride (Boster Biological Technology, Co., Ltd.), and Ly6G^+^, CD86^+^, and CD206^+^ cells were analyzed by assigning a score as follows: 1) scattered positive cells (<2%); 2) moderately abundant positive cells, diffuse or arranged in foci (2–10%); and 3) abundant positive cells, diffuse or arranged in foci (>20%) (Bianchi-Frias et al., 2019).

### RT-qPCR analysis

Total RNA was isolated from tumors using Trizol reagent (Beyotime). RNA was reverse transcribed using the SuperScript II cDNA Synthesis Kit (Takara, Japan), and real-time quantitative PCR (RT-qPCR) was performed using SuperReal PreMix Plus (TIANGEN, China). The primers are listed in the Table 1.

**TABLE 1:** Sequences of primers used to amplify cytokine (mice) and housekeeping genes.

	Forward	Reverse
**IL-1β**	5′-GCA ACT GTT CCT GAA CTC AAC T-3′	5′-ATC TTT TGG GGT CCG TCA ACT-3′
**TNF-α**	5′-CAT CTT CTC AAA ATT CGA GTG ACA A-3′	5′-TGG GAG TAGACA AGG TAC AAC CC-3′
**IL-6**	5′-CCT​TCC​TAC​CCC​AAT​TTC​CAA​T-3′	5′-AAC​GCA​CTA​GGT​TTG​CCG​AGT​A-3′
**TGF-β**	5′-GAA GGC AGA GTT CAG GGT CTT-3′	5′-GGT TCC TGT CTT TGT GGT GAA-3′
**IL-10**	5′-CTT CGA GAT CTC CGA GAT GCC TTC-3′	5′-ATT CTT CAC CTG CTC CAC GGC CTT-3′
**G-CSF**	5′-CTC​AAC​TTT​CTG​CCC​AGA​GG-3′	5′-AGC​TGG​CTT​AGG​CAC​TGT​GT-3′
**GM-CSF**	5′-GCC​ATC​AAA​GAA​GCC​CTG​AA-3′	5′-GTG​AAA​TTG​CCC​CGT​AGA​CC-3′
**IFN-γ**	5′-CTG​GAG​GAA​CTG​GCA​AAA​GG-3′	5′-CTG​GAC​CTG​TGG​GTT​GTT​GA-3′
**HPRT**	5′-TTA TGG ACA GGA CTG AAA GAC-3′	5′-GCT TTA ATG TAA TCC AGC AGG T-3′

### Flow cytometry analysis

Single cells were isolated from the tumor by suspension in collagenase I (Wuhan Servicebio Technology, Co., Ltd.) solution for 45 min at 37°C, followed by passage through a 70 μm cell strainer. Samples were incubated at 4°C for 30 min with specific fluorochrome-labeled antibodies (CD86, CD206, Ly6G, CD3, CD4, and CD8; Elabscience Biotechnology, Co., Ltd.), and at least 10,000 events were analyzed by a FACSCalibur flow cytometer (Beckman Coulter CytoFlex). Data were analyzed using FlowJo software (Tree Star).

### Statistical analysis

Statistical analyses were performed using SPSS 21.0 software, and *p*-values <0.05 were considered statistically significant. Statistical comparisons between two groups were made by the paired student’s *t*-test and a one-way ANOVA was computed to compare the differences among multiple groups. Survival analysis was conducted using the Kaplan-Meier method and the log-rank test. All data are expressed as the means ± SEM.

## Results

### IFN-γ enhances the anticancer activity of St.∆ppGpp mediated cancer immunotherapy

**FIGURE 1:**
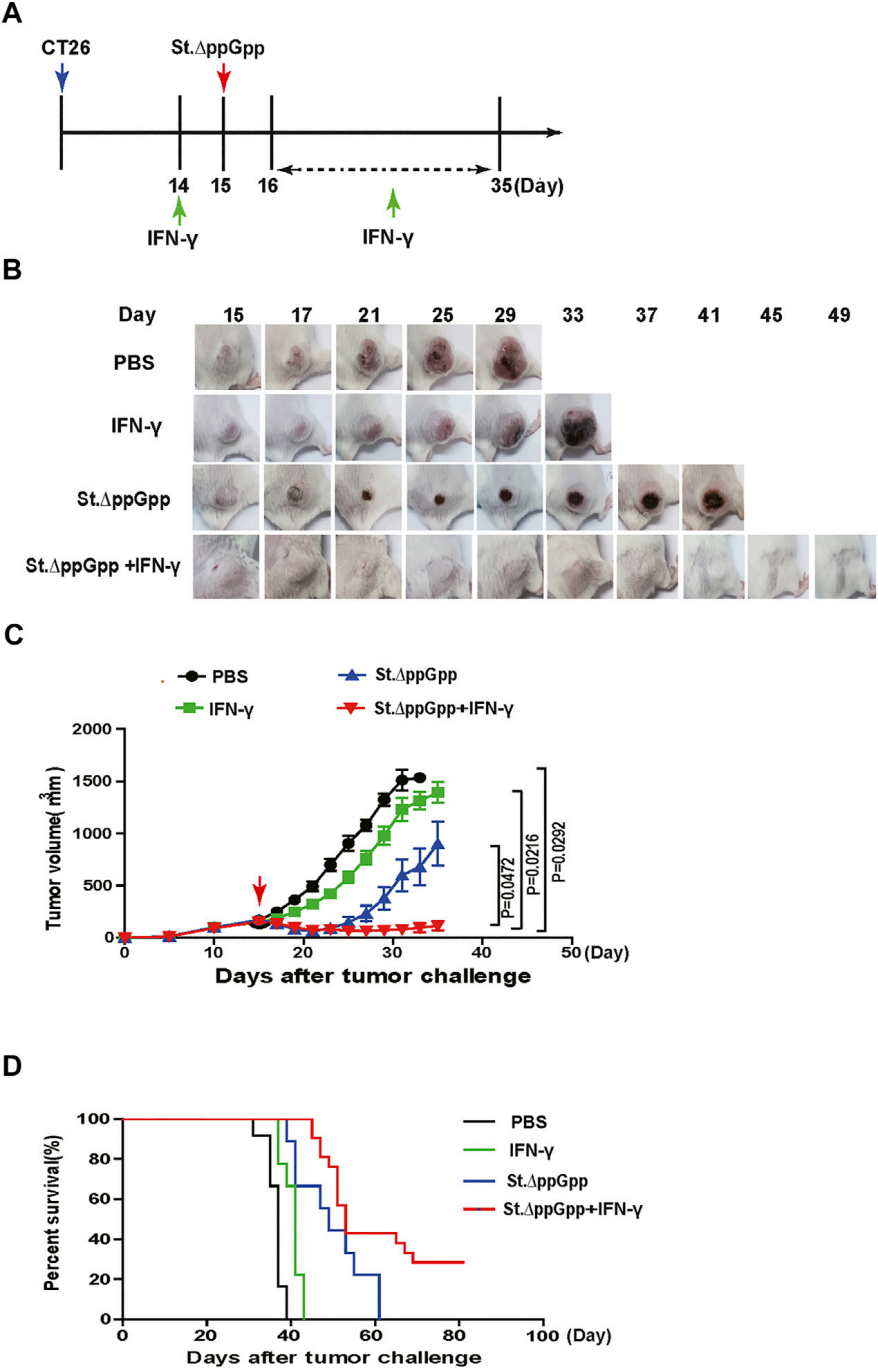
Effect of IFN-γ/St.△ppGpp combination therapy on antitumor activity. **(A)** Protocol for the treatment of mice with PBS (control), Interferon-γ (IFN-γ), St.△ppGpp, and IFN-γ plus St.△ppGpp. Male BALB/c mice (n = 9 per group, except n = 14 in IFN-γ and IFN-γ plus St.△ppGpp groups) were injected subcutaneously with CT26 cells (1 × 10^6^) on day 0. When tumors reached a volume of approximately 120 mm^3^, mice were divided into four treatment groups: PBS alone, St.△ppGpp alone, IFN-γ alone, and St.△ppGpp plus IFN-γ. Mice received 3 × 10^7^ CFU of bacteria on day 15 in the presence or absence of IFN-γ day 14, and days 16–35. **(B)** Images of tumors from representative mice from each group. **(C)** Changes in tumor volume. **(D)** Kaplan-Meier survival curves.

To determine the antitumor effects of IFN-γ/BCI combination therapy, mice were injected intraperitoneally with IFN-γ (10 mg/kg) on Day 14 post-implantation, followed by an intravenous injection of St. ΔppGpp (3 × 10^7^ CFU) on Day 15. IFN-γ injections (one injection per day) were continued up until Day 35 (Figure 1A). We found that administration of IFN-γ alone had little antitumor activity; however, the IFN-γ/St. ΔppGpp combination led to marked tumor regression (Figures 1B,C) and a higher survival rate (Figure 1D) compared with either IFN-γ or St. ΔppGpp-alone, suggesting the IFN-γ/St. ΔppGpp combination has a synergistic antitumor effect.

### IFN-γ increases the survivor of tumor-colonizing bacteria

**FIGURE 2:**
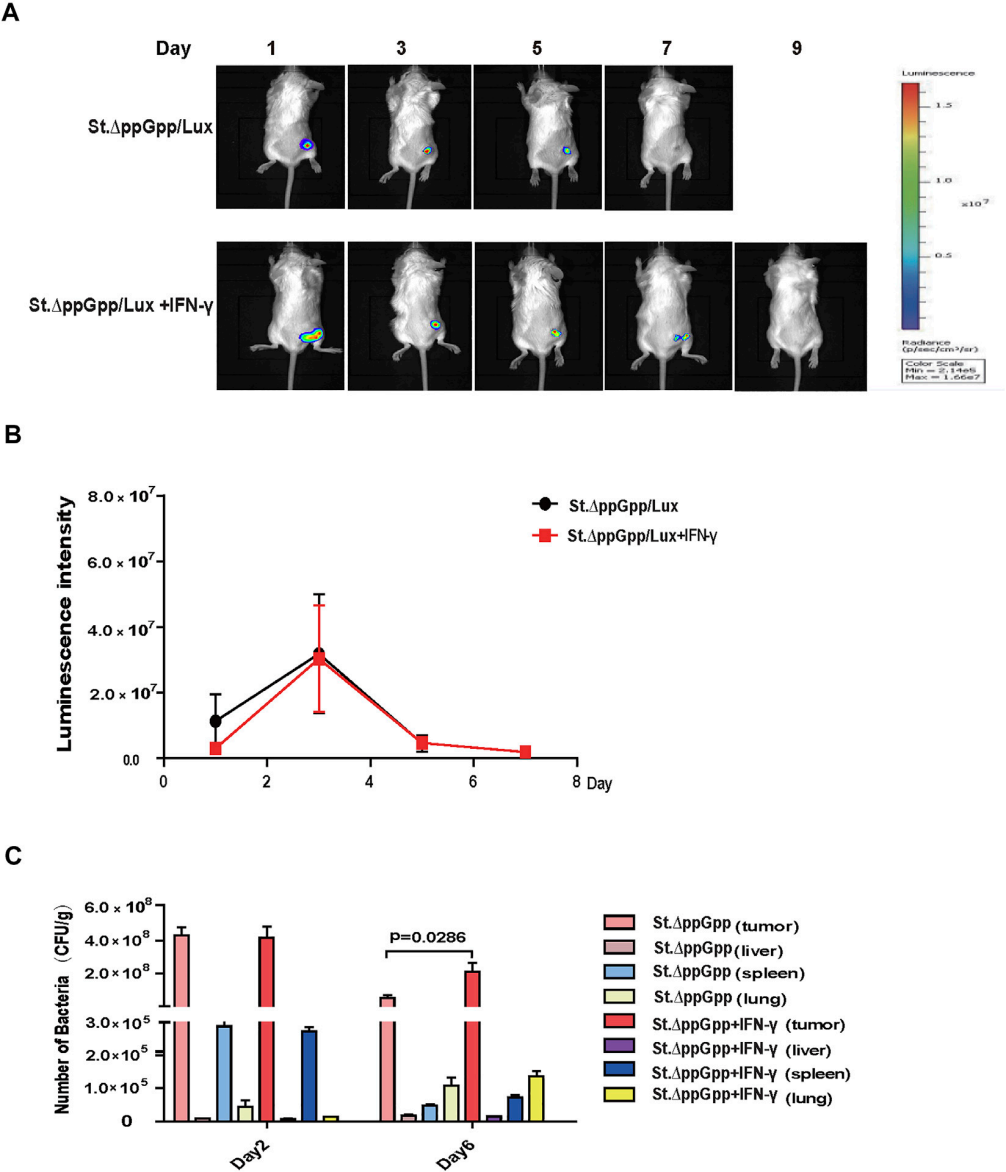
Effect of IFN-γ/St.△ppGpp combination therapy on tumor-colonizing bacteria. **(A)** Non-invasive monitoring of bacterial bioluminescence for 9 days and **(B)** quantitative. **(C)** Bacterial counts in an isolated organ of tumor-bearing mice on days 2 and 6.

The efficacy with which bacteria kill tumor cells is associated with their survival within the tumor; thus, we used St. ΔppGpp -Lux-expressing bioluminescent bacteria to assess survival of tumor-colonizing bacteria during IFN-γ/St. ΔppGpp combination therapy. IFN-γ administration increased the survival of tumor-colonizing St. ΔppGpp-Lux from 5 to 7 days (Figures 2A,B, as determined by bioluminescence imaging. To assess the effect of IFN-γ/St. ΔppGpp on tumor targeting by bacteria, we isolated tumor, lung, liver, and spleen from tumor-bearing mice injected with St. ΔppGpp, and counted colonizing bacteria on Days 2 and 6. In agreement with the results of bioluminescence imaging, IFN-γ administration led to a significant increase in accumulation of tumor-colonizing bacteria on Day 6 (Figure 2C), suggesting that IFN-γ increases the survival of tumor-colonizing bacteria, thereby increasing the efficacy of tumor cell killing.

### Toxicity of IFN-γ/St.∆ppGpp combination therapy

**FIGURE 3:**
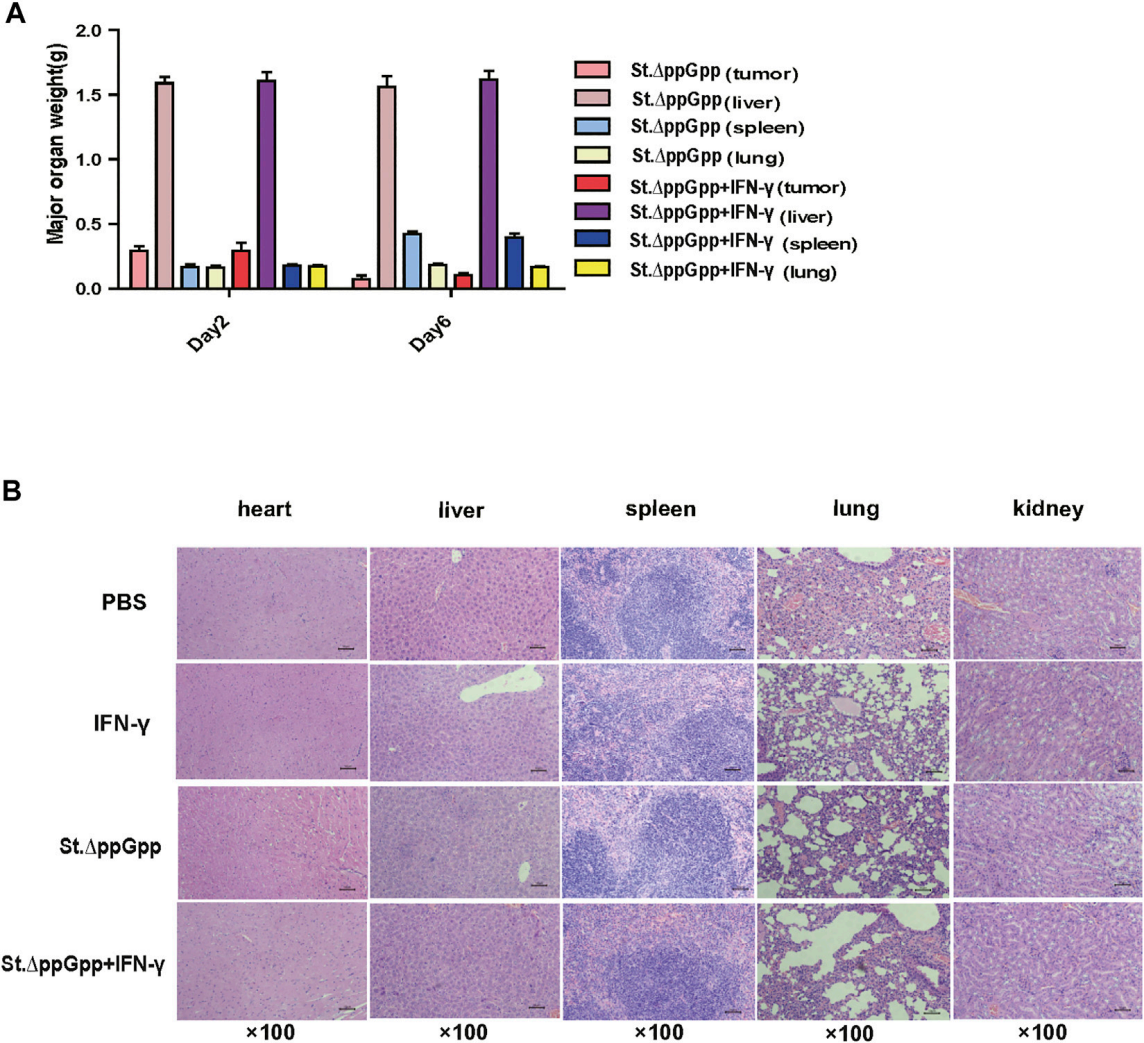
Toxicity evaluation of IFN-γ/St.△ppGpp combination therapy. **(A)** Organ weight on day 2 and 6. **(B)** H&E staining for isolated organs of tumor-bearing mice on day 6 after BCI treatment.

To evaluate the safety of IFN-γ/St.∆ppGpp combination therapy, heart, lung, liver, spleen, and kidney were removed from tumor-bearing mice after the bacterial injection on Day 6, weighed, and stained with H&E. There was no difference in the weight of organs from treated and control mice (Figure 3A), nor any signs of significant toxicity, including steatosis, inflammatory infiltrates, or fibrosis (Figure 3B).

IFN-γ/St.∆ppGpp combination therapy increases the recruitment of tumor-infiltrating macrophages and decreases the recruitment of tumor-infiltrating neutrophils.

**FIGURE 4:**
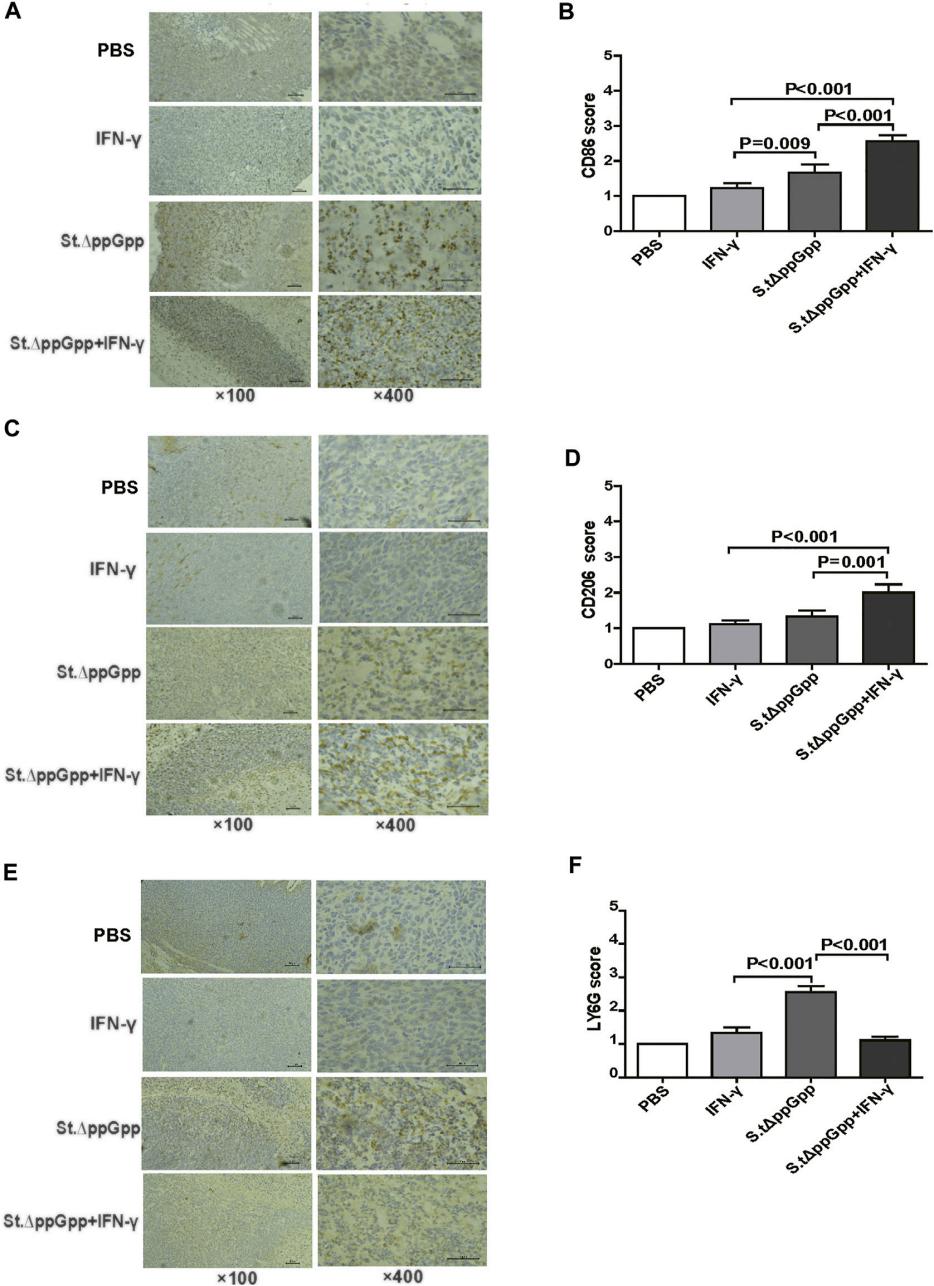
Effect of IFN-γ/St.△ppGpp combination therapy on tumor-infiltrating neutrophils and macrophages in tumor-bearing mice. Mice were sacrificed, and excised tumors were stained with hematoxylin and eosin and subjected to immunohistochemical staining (CD86 antibody) for 48 h after injection with or without SL. ΔppGpp (n = 3). The sections were visualized with 3, 3′-diaminobenzidine tetrahydrochloride and Ly6G^+^, CD86^+^, and CD206^+^ cells were analyzed by assigning a score as follows: (I) scattered positive cells (<2%); ii) moderately abundant positive cells, diffuse or arranged in foci (2–10%); and iii) abundant positive cells, diffuse or arranged in foci (>20%). **(A)**. The expression of CD86 was significantly higher in mice from the SL. ΔppGpp plus IFN-γ group than in those from the SLΔppGpp group **(B)**. Excised tumors were stained with CD206 antibody for immunohistochemical staining (n = 3) **(C)**. The expression of CD206 was significantly higher in mice from the SL. ΔppGpp plus IFN-γ group than in those from the SLΔppGpp group **(D)**. Excised tumors were stained with LY6G antibody for immunohistochemical staining (n = 3) **(E)**. The expression of LY6G was significantly higher in mice from the SL. ΔppGpp plus IFN-γ group than in those from the SLΔppGpp group **(F)**.

Tumor-associated macrophages (TAMs) are classified as M1-type (antitumorigenesis) and M2-type (protumorigenesis). A shift from M2 to M1 is induced by BCI, which promotes tumor regression (Zheng et al., 2017). Thus, we next determined the effect of IFN-γ/St.∆ppGpp therapy on TAM polarization. Tumor tissues were stained for CD86 (to detect M1-type macrophages) and CD206 (to detect M2-type macrophages), and subjected to immunohistochemical analyses (IHC). We found that IFN-γ/St.∆ppGpp increased the numbers of both CD86^+^ (Figures 4A,B and CD206^+^ (Figures 4C,D) cells in tumor sections when compared with St.∆ppGpp-alone, suggesting that although IFN-γ/St.∆ppGpp did not affect macrophage polarization, it increased the number of M1-type TAMs.

Neutrophils are the first immune cells recruited to the tumor after bacterial colonization; these immune cells inhibit bacterial invasion from the necrotic zone to the viable cancer cell zone, thereby preventing further tumor cell killing (Westphal et al., 2008a). Therefore, blocking tumor-infiltrating neutrophil recruitment should promote bacteria-mediated tumor cell killing. To investigate whether IFN-γ-mediated increases in survival of tumor-colonizing bacteria affect recruitment of tumor-infiltrating neutrophils, we stained tumor tissues with LY6G (to detect neutrophil). IHC revealed that the proportion of LY6G^+^ cells in tumor sections from the IFN-γ/St.∆ppGpp group was lower than that in sections from the St.∆ppGpp-alone group (Figures 4E,F). This suggests that IFN-γ blocks tumor-infiltrating neutrophil recruitment induced by BCI and favors the survival of tumor-colonizing bacteria.

### Effect of IFN-γ/St.∆ppGpp on tumor inflammatory cytokine levels

**FIGURE 5:**
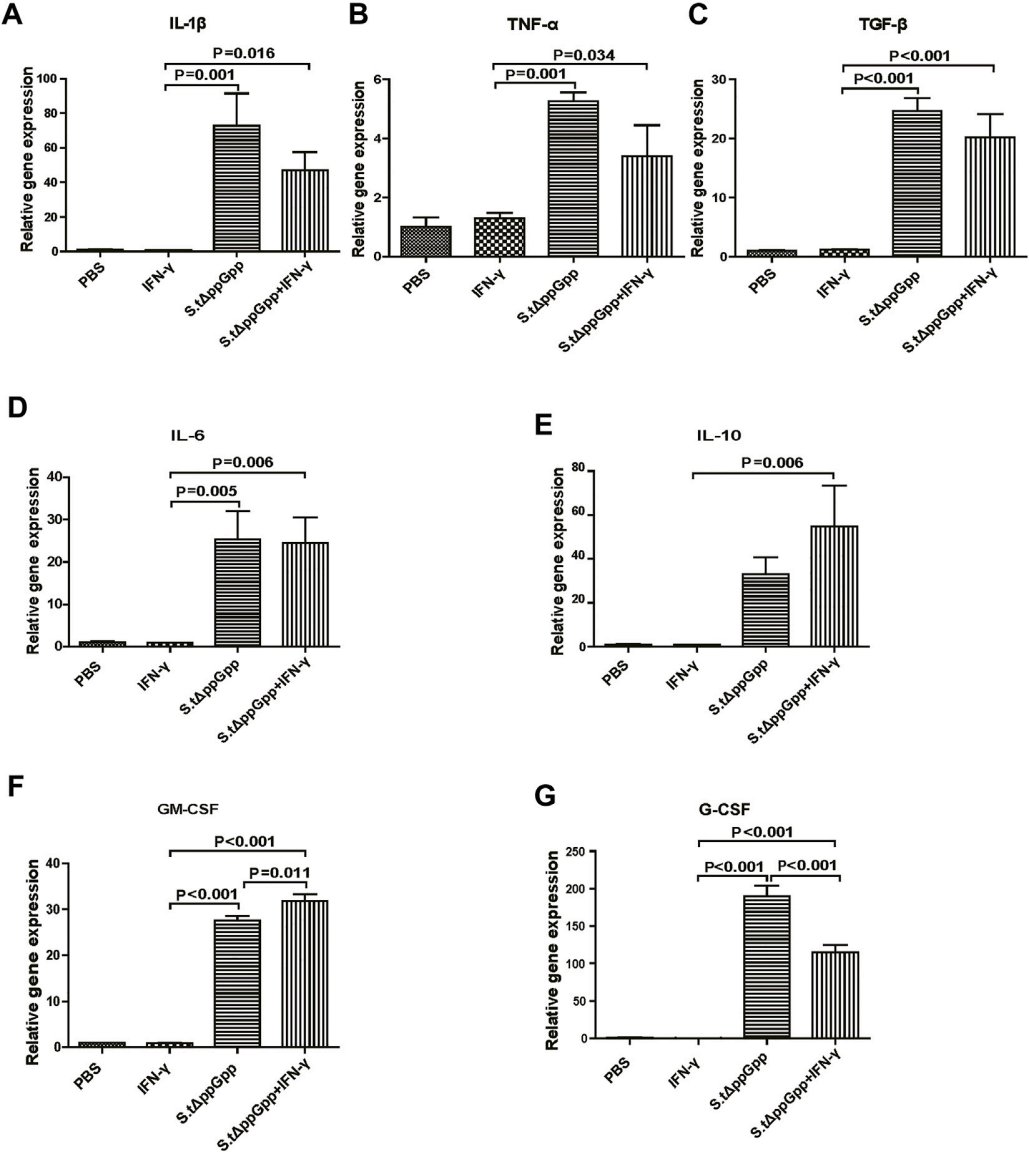
Effect of IFN-γ/St.△ppGpp combination therapy on inflammatory cytokines. Tumor tissue was isolated from CT-26 tumor-bearing mice at day 2 post-bacterial injection (n = 3 mice/group), and mRNA expression of inflammatory cytokines, including *Il-1β* **(A)**, *Tnf-α* **(B)**, *Tgf-β* **(C)**, *Il-6* **(D)**, *Il-10* **(E)**, *Gm-csf* **(F)**, and *Gm-csf* **(G)**, was measured by qRT-PCR. *Hprt* was used as a housekeeping gene.

In response to tumor invasion by bacteria, M1-type TAMs secrete proinflammatory cytokines; tumor-infiltrating M2 macrophages (protumorigenesis) secrete anti-inflammatory cytokines that regulate the tumor-suppressive efficacy of BCI. To determine the effects of IFN-γ/St.∆ppGpp on tumor inflammatory cytokines, we removed the tumor tissue from CT26 tumor-bearing mice and used qRT-PCR to measure *Il-1β*, *Tnf-α*, *Tgf-β*, *Il-6*, *Il-10*, *Gm-CSF*, and *G-CSF*. We found no significant difference in expression of proinflammatory cytokines *Il-1β* and *Tnf-α* (Figures 5A,B, and anti-inflammatory cytokines *Il-6*, *Tgf-β*, and *Il-10* (Figure 5C–E), between the IFN-γ/St.∆ppGpp and St.∆ppGpp-alone groups. We believe that this was due to an increase in both M1-and M2-type TAMs in the IFN-γ/St.∆ppGpp group, which was confirmed by IHC staining. Moreover, expression of *Gm-CSF* increased in the IFN-γ/St.∆ppGpp group (Figure 5F), whereas expression of *G-CSF* decreased (Figure 5G). Again, this may be associated with an increase in the number of tumor-infiltrating macrophages and a decrease in the tumor-infiltrating neutrophils, as shown by IHC analysis.

### Effect of IFN-γ/St.∆ppGpp on tumor-infiltrating immune cells

**FIGURE 6:**
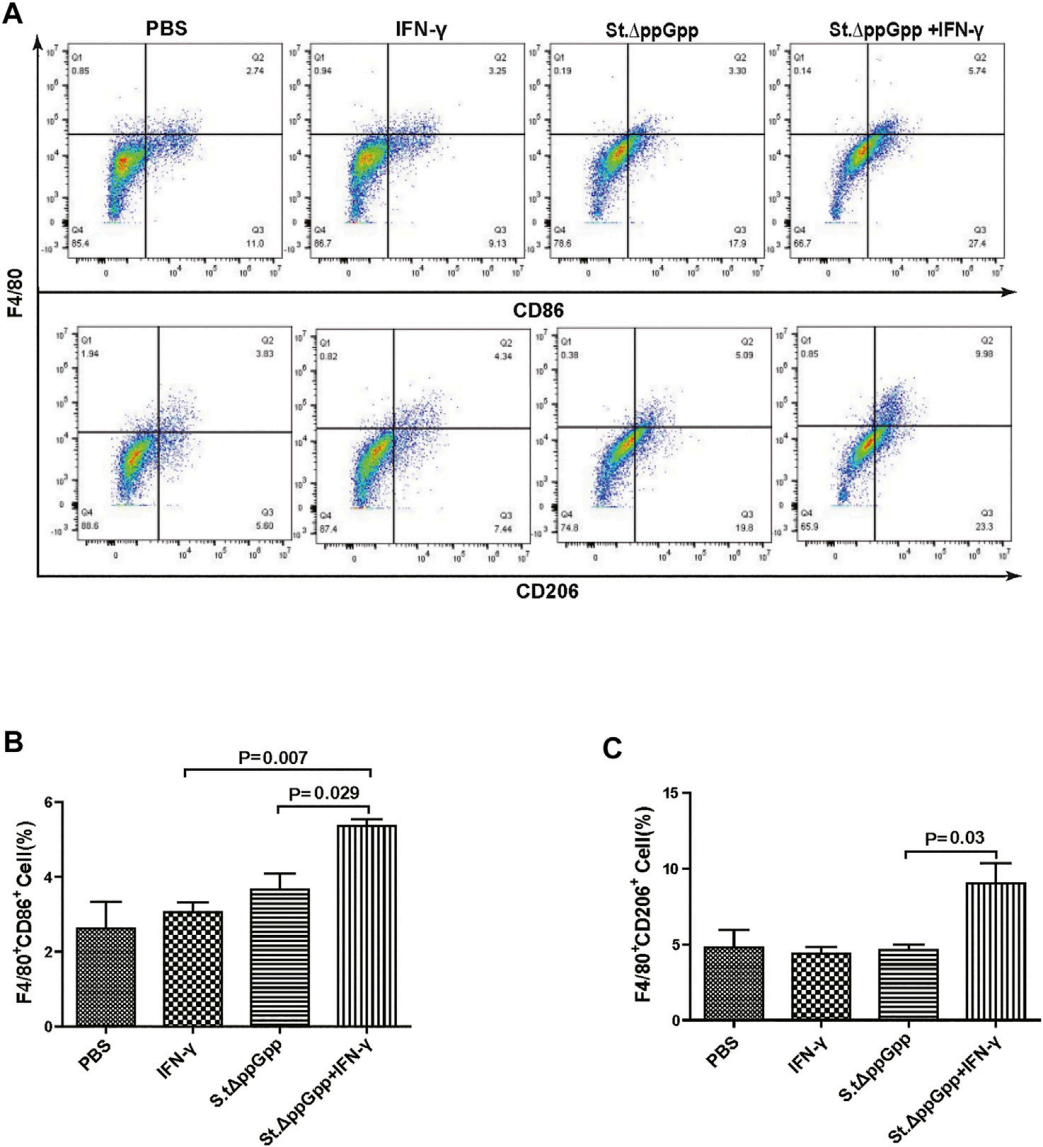
Effect of IFN-γ/St.△ppGpp combination therapy on M1 and M2 macrophage. Samples were prepared from CT26 tumor-bearing mice at day 2 post-bacterial injection (n = 5 mice/group). **(A)** Samples were double-stained with F4/80 (macrophage marker), and CD86 (M1-type macrophage) or CD206 (M2-type macrophage) and analyzed by Flow Cytometry (A) and graph **(B,C)**.

**FIGURE 7:**
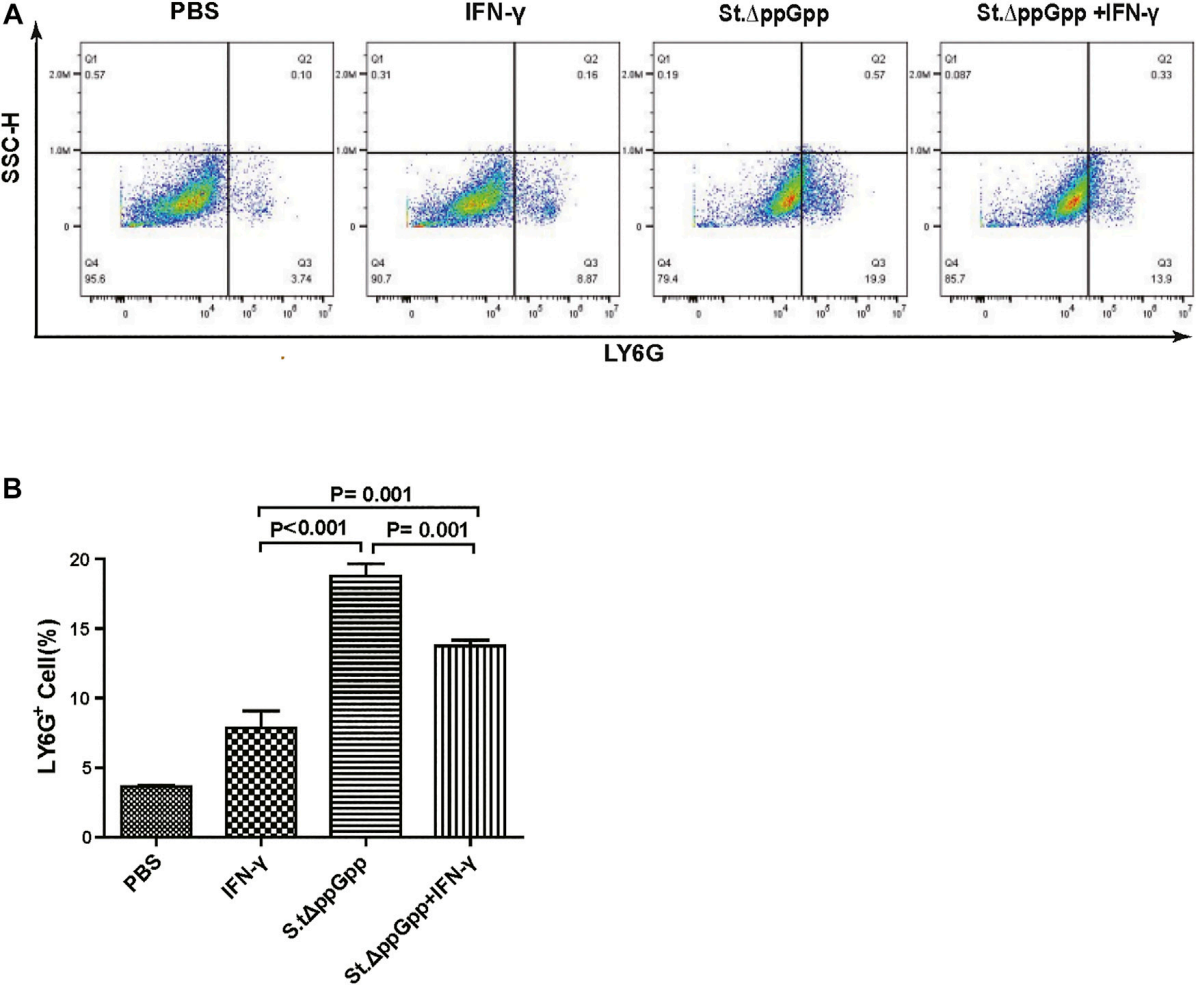
Effect of IFN-γ/St.△ppGpp combination therapy on tumor-infiltrating neutrophils. Samples were prepared from CT26 tumor-bearing mice at day 2 post-bacterial injection (n = 5 mice/group). **(A)** Samples were stained with LY6G (neutrophil marker) and analyzed by Flow Cytometry **(A)** and graph **(B)**.

**FIGURE 8:**
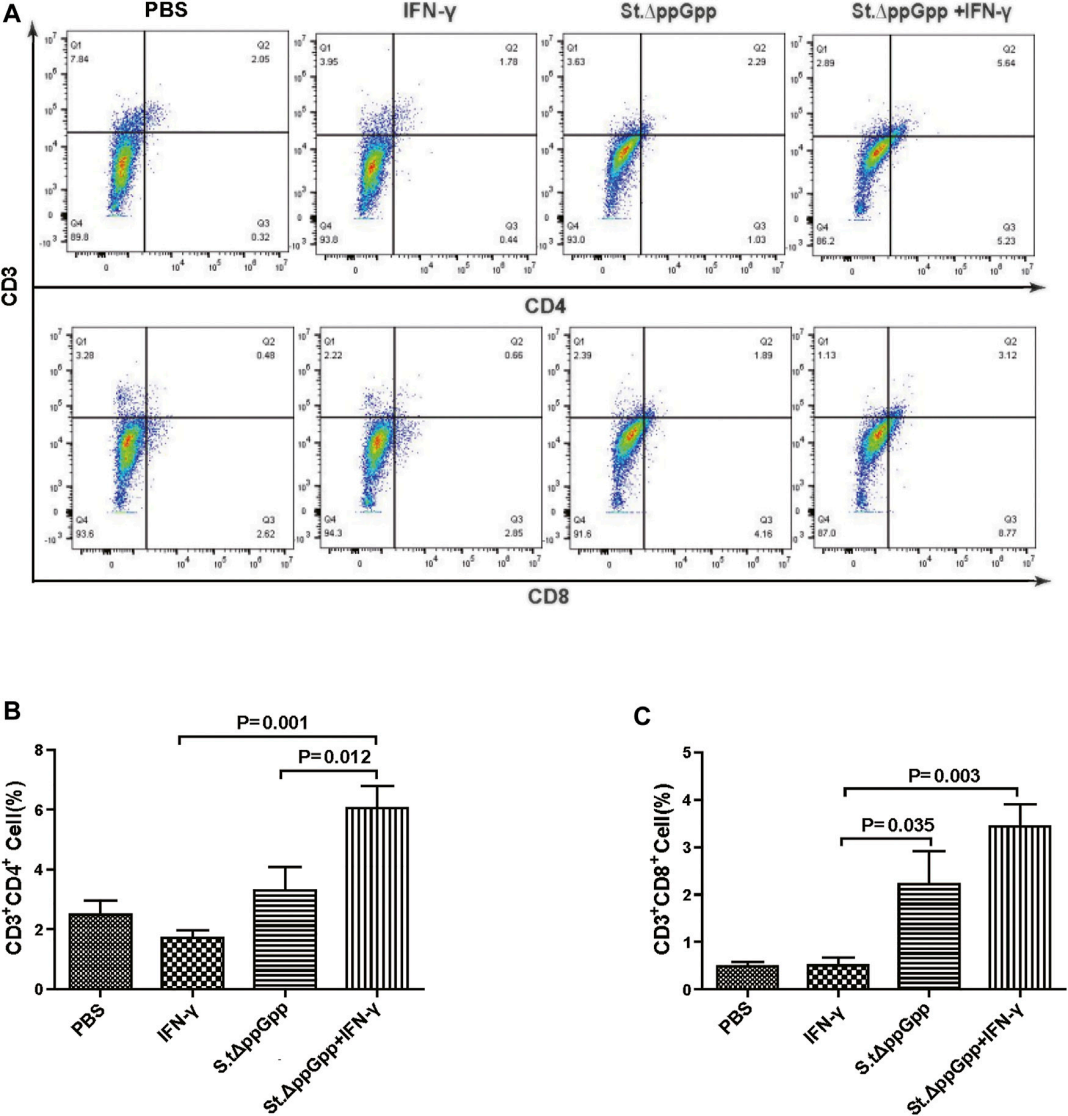
Effect of IFN-γ/St.△ppGpp combination therapy on CD8 and CD4 T cell. Samples were prepared from CT26 tumor-bearing mice at day 2 post-bacterial injection (n = 5 mice/group). **(A)** Samples were double stained with CD3 (T cell marker), and CD4 (CD4 T cell marker) or CD8 (CD8 T cell marker) and analyzed by Flow Cytometry (A) and graph **(B,C)**.

BCI-induced tumor regression is not only mediated by tumor cell killing by bacteria themselves, but also by host immune cell-dependent responses by neutrophils, macrophages, and CD4^+^ and CD8^+^ T cells (Lee et al., 2008; Barak et al., 2010; Lee et al., 2011a; Lee et al., 2011b). Therefore, to examine the role of host immune cell-dependent responses in the antitumor therapeutic effects of IFN-γ/St.∆ppGpp, we performed Flow cytometry analysis to measure the immune cell populations. Tumor tissues were removed from CT26 tumor-bearing mice and double-stained with F4/80 and CD86 (to detect M1-type macrophages) or with F4/80 and CD206 (to detect M2-type macrophages). We found that the numbers of both F4/80^+^CD86^+^(M1-type macrophages) and F4/80^+^CD206^+^ (M1-type macrophages) in the IFN-γ/St.∆ppGpp group were higher than in the St.∆ppGpp-alone group (Figures 6A,C). This finding supports the results of ICH staining, which clearly showed that IFN-γ/St.∆ppGpp increased both the CD86^+^ and CD206^+^ fractions in tumor sections (Figures 4A,D). Consistent with the observations from IHC staining (Figures 4E,F), we found that IFN-γ decreased the numbers of St.∆ppGpp-induced tumor-infiltrating neutrophils (LY6G^+^) (Figures 7A,B. We then assessed the tumor-infiltrating T cell population by double staining tumor tissue with CD3 and CD4 (to detect CD4^+^ T cells) or CD3 and CD8 (to detect CD8^+^ T cells). The results (Figures 8A,C) showed that IFN-γ/St

.∆ppGpp increased recruitment of both CD4^+^ and CD8^+^T cells. Given that M1-type macrophage, and CD4^+^ and CD8^+^ T cells, exert antitumor effects, these results suggest that IFN-γ enhanced St.∆ppGpp-priming of this immune cell response, leading to further tumor suppression.

## Discussion

Tumor targeting by oncolytic *Salmonella* kills tumor cells directly *via* production of massive amounts of nitric oxide (NO), which induce tumor cell apoptosis (Barak et al., 2010) and increase expression of autophagy-related proteins (Beclin, LC3) to induce tumor cell autophagy (Lee et al., 2014). Here, we demonstrated that IFN-γ/St.∆ppGpp combination therapy significantly inhibited tumor growth in mice, resulting in a higher survival rate in tumor-bearing mice compared with either IFN-γ or St.∆ppGpp alone. Moreover, we observed that the IFN-γ/St.∆ppGpp combination increased the number and survival of tumor-colonizing bacteria, which are associated with tumor cell killing efficacy. This observation is supported by the previous finding that survival of tumor-colonizing St.∆ppGpp was prolonged by ionizing radiation, which enhances the antitumor effect of BCI (Liu et al., 2016). As the earliest immune cell recruited in response to bacterial tumor invasion, tumor-infiltrating neutrophils inhibit tumor-colonizing bacterial migration from the necrotic to the viable tumor cell zone, which limits the tumor cell killing effects. The results of HCI staining and Flow Cytometry show that IFN-γ significantly blocks St.∆ppGpp-induced recruitment of tumor-infiltrating neutrophils, which supports Westphal’s observation that antibody-mediated depletion of neutrophils promotes bacterial antitumor activity by facilitating bacterial tumor colonization (Westphal et al., 2008b), as well as those of another study showing that IFN-γ attenuates infiltration of neutrophils into the tumor microenvironment (Manoury-Schwartz et al., 1997; Irmler et al., 2007). Neutrophils had three possible fates in response to inflammation: forward migration to the inflammatory site, apoptosis, and reverse migration from the inflammatory site, which is associated with the phosphorylation of AKT and ERK (Zhang et al., 2020; Zhang et al., 2021). We guessed IFN-γ attenuates infiltration of neutrophils by apoptosis or reverse migration.

IFN-γ-mediated enhancement of bacteria-specific tumor targeting did not cause any toxicity in the heart, liver, spleen, lung, or kidney, indicating that IFN-γ increased accumulation of bacteria in tumors, but not bacterial virulence.

The tumor-suppressive effects of bacteria are not only direct; they are also mediated by host immune responses. Tumor-infiltrating M1-type macrophages (anti-tumorigenesis) secrete proinflammatory cytokines (e.g., IL-1β and TNF-α), whereas tumor-infiltrating M2-type macrophages (pro-tumorigenesis) secrete anti-inflammatory cytokines (e.g., IL-6 and IL-10) (Grille et al., 2014; Chirullo et al., 2015). M2-to-M1 conversion is named “macrophage polarization,” resulting in conversion of a high immunosuppressive tumor microenvironment into an immunogenic one (Lin et al., 2019; Pan et al., 2020; Wang et al., 2021); thus, targeting macrophage conversion is a potential therapeutic strategy. Zheng et al. reported that FlaB-secreting *Salmonella* enhance host antitumor immune response *via* TLR5-mediated M2-to-M1 conversion (Zheng et al., 2017). Herein, we observed an increase in both M1-type and M2-type macrophages in the IFN-γ/St.∆ppGpp group, suggesting that non-M2-to-M1 shifting-mediated enhancement of M1-type macrophage recruitment also contributes to the antitumor effects of BCI. This increase in both types of macrophages resulted in no changes in the tumor inflammatory cytokine profile. The prior study indicated that the reduction of a tumor-infiltrating neutrophil by CD11b agonist resulted in an increase in the number of CD8^+^ and CD4^+^ T cells (Panni et al., 2019). Although there is no significance in St.∆ppGpp versus IFN-γ/St.∆ppGpp in tumor inflammatory cytokine profile, we thought anti-inflammatory prevails over the pro-inflammatory, which perhaps associated with a decreased tumor-infiltrating neutrophil recruitment, and this effect thereby boosted the infiltration of CD8^+^ and CD4^+^ T cell.

T cell infiltration and their associated immune responses are critical for antitumor efficacy and are regulated by TAMs and neutrophils (dependent on their phenotype) (Fridlender et al., 2009; Jackaman et al., 2016; Jackaman et al., 2017; Garrido-Martin et al., 2020; Pan et al., 2020). Herein, we observed an increase in the CD4^+^ and CD8^+^ T cell population in the IFN-γ/St.∆ppGpp group relative to the St.∆ppGpp-alone group, implying that infiltration by CD4^+^ and CD8^+^ T cells is associated with a decrease in tumor-infiltrating neutrophils and an increase in tumor-infiltrating M1-type macrophages, leading to further tumor suppression. A more in-depth understanding of the mechanisms that regulate neutrophil and M1-type macrophages in response to therapeutic bacteria is needed to develop combination therapy to enhance the efficacy of BCI.

## Conclusion

In summary, we demonstrated that IFN-γ enhanced the tumor cell killing efficacy of attenuated *Salmonella* by prolonging bacterial survival *via* inhibition of tumor-infiltrating neutrophil recruitment, and by increasing *Salmonella*-stimulated immune responses *via* activation of tumor infiltration by M1-like macrophages and CD4^+^ and CD8^+^ T cells. Thus, IFN-γ/St.∆ppGpp combination therapy is a promising approach to cancer treatment.

## Data Availability

The original contributions presented in the study are included in the article, further inquiries can be directed to the corresponding authors.
